# Bayesian causal inference reveals declined proprioception, increased integration bias underlie older adults’ stronger visual bias in hand position perception

**DOI:** 10.1038/s41598-026-45797-3

**Published:** 2026-04-12

**Authors:** Naoki Kuroda, Yoshiyuki Sato, Shinya Harada, Ryo Teraoka, Wataru Teramoto

**Affiliations:** 1 Research Organization of Open Innovation and Collaboration, Ritsumeikan University, 2-150 Iwakura-cho, Ibaraki, Osaka 567-8570 Japan; 2 Japan Society for the Promotion of Science, Kojimachi Business Center Building, 5-3-1 Kojimachi, Chiyoda-ku, Tokyo, 102-0083 Japan; 3 Faculty of Humanities and Social Sciences, Kumamoto University, 2-40-1 Kurokami, Chuo-ku, Kumamoto, 860-8555 Japan; 4 Department of Human Informatics, Faculty of Human Informatics, Aichi Shukutoku University, 2-9 Katahira, Nagakute, Aichi 480-1197 Japan; 5 Graduate School of Engineering, Muroran Institute of Technology, 27-1 Mizumoto-cho, Muroran, Hokkaido 050-8585 Japan

**Keywords:** Hand position perception, Visuo-proprioceptive integration, Aging, Bayesian causal inference model, Integration strategy, Neuroscience, Psychology, Psychology

## Abstract

Self-localization is fundamental to bodily self-consciousness across the lifespan. Humans estimate body-part position by integrating afferent signals such as vision and proprioception. Rubber and mirror hand illusions highlight the dominant role of vision in hand position perception. Although older adults rely more heavily on visual information, the computational mechanisms underlying age-related increases in visual bias remain unclear. Here, we examined age-related changes in visuo-proprioceptive integration using a Bayesian causal inference (BCI) model. Two experiments introduced spatial discrepancies between visual and proprioceptive hand positions to manipulate the likelihood of integration. Participants reached toward a target after the visual hand disappeared, allowing the BCI model to estimate sensory reliabilities and the prior probability of a common cause ($\:{p}_{common}$). Decision-making strategies were also compared within the BCI framework. Older adults exhibited reduced proprioceptive reliability and a higher $\:{p}_{common}$, indicating a stronger tendency to infer a shared source for visual and proprioceptive signals. No age-related differences were observed in decision-making strategy. These findings suggest that age-related visual bias reflects changes not only in sensory reliability but also in causal inference during multisensory integration.

**Supplementary Information:**

The online version contains supplementary material available at 10.1038/s41598-026-45797-3.

## Introduction

The body underpins action and interaction throughout life, and accurate localization of body parts is essential for effective engagement with the environment. The brain estimates body position by integrating visual and proprioceptive signals, as illustrated by the rubber hand illusion (RHI), in which synchronous stroking^1^ or movement^2^ of a visual fake and hidden real hand induces a sense of ownership of the fake hand and shifts the perceived location of the real hand toward the fake one^1^. This perceptual displacement also influences goal-directed actions^3^, demonstrating the strong impact of visual input on perception and action.

Although early work reported little effect of aging on perceived hand position in the RHI^4–6^, recent studies have shown that visual information increasingly dominates proprioception in older adults^7–10^. This age-related visual bias appears across paradigms such as the mirror hand illusion^9–11^ and projected-hand illusion^3,7,8^, which similarly introduce visuo-proprioceptive (VP) mismatches. Despite converging evidence for stronger visual bias with age, the computational mechanisms underlying these changes remain unclear.

The BCI model provides a powerful framework for understanding multisensory integration and can explain perceptual phenomena associated with the RHI and related illusions^12–17^. Critically, the BCI model captures not only sensory integration—how signals are combined based on their relative reliabilities, which has also been explained by the maximum likelihood estimation model (or forced-fusion model)^18^—but also causal inference, that is, how the brain determines whether sensory signals share a common cause. Although many studies have reported enhanced multisensory integration with aging^19,20^, it remains unclear how aging affects the prior belief in a common cause, a core component uniquely formalized in the BCI framework. Recent studies have demonstrated the use of this model to quantify age-related changes in VP integration^7,8^. However, because those studies employed nearly identical virtual reality setups and tasks, the generalizability of their findings across experimental contexts remains uncertain.

In bimodal localization tasks, the BCI model estimates sensory-event locations by considering noisy measurements and causal structure. It combines signals according to their relative reliabilities and the prior probability—shaped by experience—that they arise from a common source. Based on the inferred causal relationship, the model determines whether signals should be integrated or treated separately, allowing causal inference and sensory integration to be modeled within a single computational framework^14^. Importantly, this framework enables age-related multisensory differences to be decomposed into changes in unisensory reliability, causal inference, and decision-level computations.

Decision-making strategies play a critical role in how the BCI models generate behavioral outputs. Proposed strategies include model averaging (MA), model selection (MS), and probability matching (PM)^21^. MA minimizes errors, whereas MS selects the most probable causal structure; PM—although less efficient—can be advantageous for long-term environmental exploration^21^. Older adults show different strategies in audiovisual integration, with younger adults tending to use PM and older adults relying more on MS^22^. However, whether similar age-related differences in decision strategies occur in VP integration for hand position perception has not yet been investigated.

This study addressed these gaps by examining age-related changes in VP integration for hand position perception using a BCI modeling framework. We focused on three mechanisms that may underlie the stronger visual bias observed in older adults. The first mechanism involves age-related reductions in unisensory reliability, as visual^23^ and proprioceptive^24^ functions become noisier with age, shifting the relative weighting of each signal. The second mechanism concerns changes in causal priors. Work combining the RHI with BCI modeling has shown that increasing proprioceptive noise in younger adults elevates the prior probability of a common cause between the seen rubber hand and felt real hand, intensifying the illusion without altering sensory reliabilities^25^. Thus, reduced proprioceptive precision in older adults may similarly increase the prior probability for multisensory integration, either alongside or independent of reliability changes. The third mechanism involves age-related differences in decision-making strategies. Alternatively, it is possible that age-related differences cannot be fully explained by these mechanisms alone and that the standard BCI model may not fully capture older adults’ behavioral patterns.

**Fig. 1:**
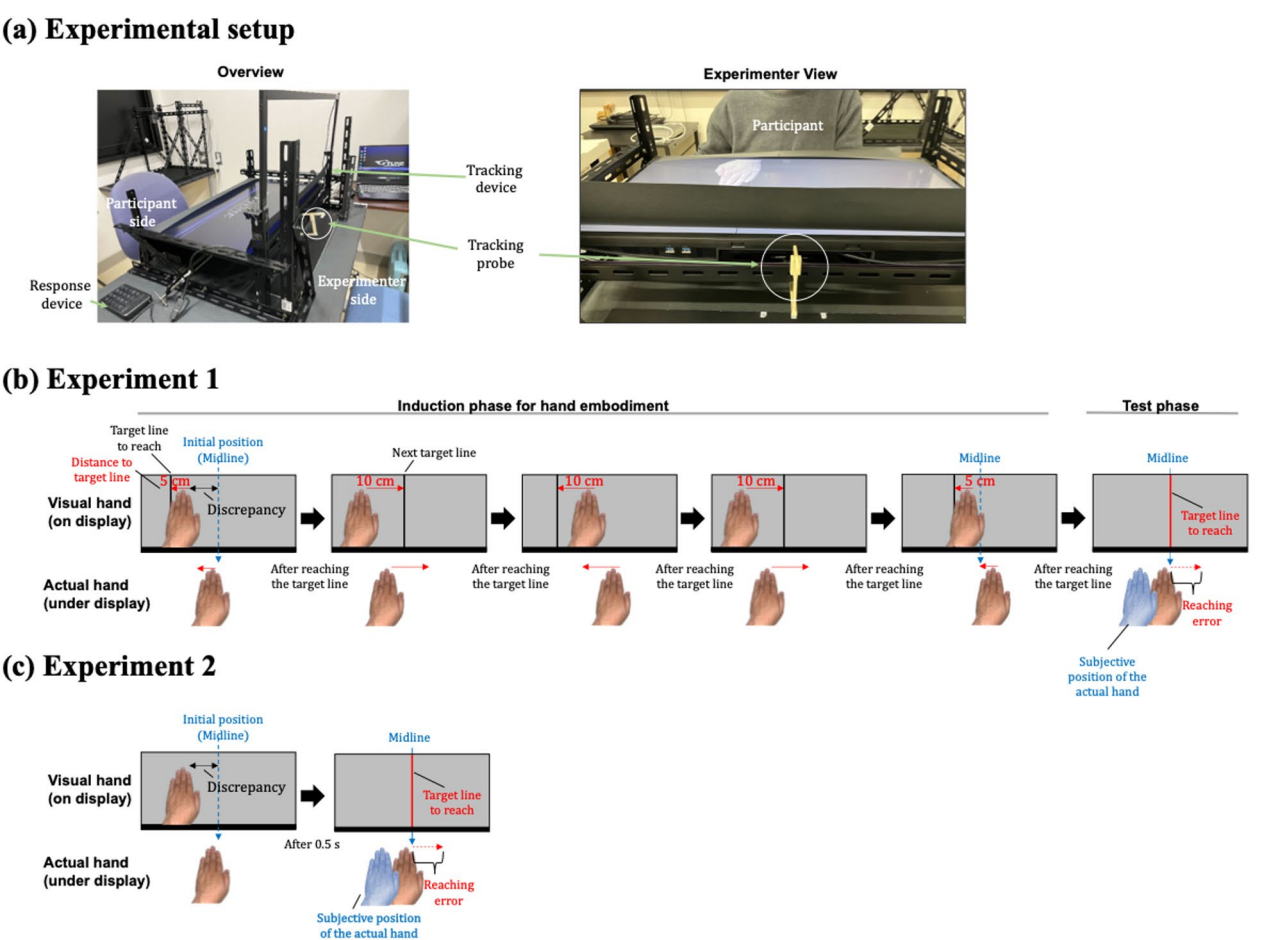
Experiment setup and procedure. (**a**) Apparatus used in Experiments 1 and 2. A large, upward-facing display was mounted on metal brackets 8 cm above the table. A slide rail was positioned in the gap beneath the display, with a movable board supporting the participant’s left hand while allowing lateral hand movement out of view. The lateral position of the left index finger was recorded using an infrared tracking device. A black paper covered the gap between the participant’s upper limb and the display, creating the impression that the real hand was projected onto the screen. (**b**, **c**) Procedure after fixation offset. The real and visual hands are illustrated in separate lower and upper panels only for visual clarity. In the actual setup, the real and visual hands were matched in depth and discrepancies were introduced only in the lateral direction. In Experiment 1, the visual and actual hands moved laterally in near synchrony. During the induction phase, a black probe line appeared, and participants aligned the visual index finger with the line. Each successful alignment triggered a new line on the opposite side; this sequence was repeated five times to induce ownership of the visual hand. The final line appeared at the original visual-hand position, returning the real hand to the midsagittal plane without participants’ explicit awareness. Immediately afterward, both the black line and hand image disappeared, and a red target line appeared at the midsagittal plane. Participants then aligned their real index finger with the target as quickly and accurately as possible. Although the real hand was already aligned with the target, the preceding visual manipulation induced a perceived displacement of hand position (illustrated by blue hand). Approximately 20% of trials were catch trials, wherein the true hand position was physically shifted by ± 5 cm relative to the red target to prevent detection of the manipulation. In Experiment 2, the embodiment phase with black-line movement was omitted. Instead, a static visual hand was presented for 0.5 s before being replaced by the red target line. The dashed blue line at the midline was not visible to participants.

We evaluated these possible mechanisms by conducting two experiments using a video-projection system (Fig. 1a) to display a static pre-recorded image of each participant’s hand, which moved in real time with their actual hand movements. Following Teramoto^9^, Experiment 1 included an induction phase to evoke ownership of the visual hand and measured how VP mismatches influenced reaching. Experiment 2 omitted the induction phase, following Fang et al.^16^, to reduce variability in ownership susceptibility and its persistence across age groups. Removing the embodiment phase should clarify intrinsic age-related differences in causal priors, as this phase may otherwise artificially elevate the prior probability of a common cause through, for example, an adaptation to the displaced visual feedback.

## Results

In Experiment 1, participants repeatedly aligned the index finger of their displaced visual hand with a black probe line to facilitate embodiment of the hand (Fig. 1b). In Experiment 2, the visual hand was presented briefly (0.5 s), with no embodiment procedure (Fig. 1c). In both experiments, participants were instructed to reach a visual target line with their invisible (proprioceptive) hand immediately after the visual hand disappeared. The spatial discrepancy between the visual and real hands (measured as the distance between the visual and actual index finger positions) was systematically manipulated across five levels: − 14, − 7, 0, 7, and 14 cm. The reaching error in each trial was calculated as the horizontal distance between the reach endpoint and target line, with positive and negative reaching errors respectively indicating leftward and rightward shifts in perceived hand position.

### Experiment 1

#### Behavioral data

**Fig. 2:**
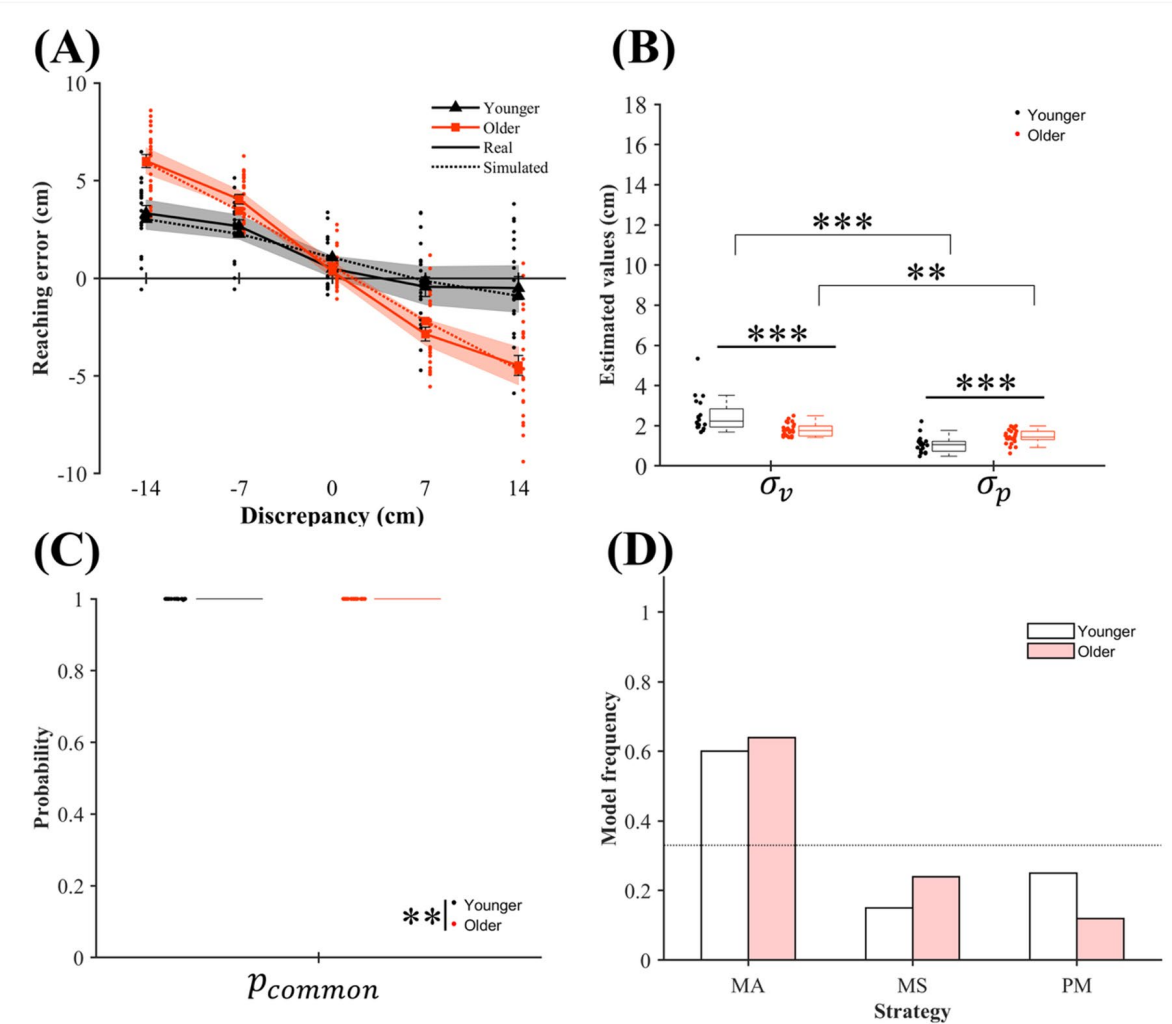
Behavioral results, estimated parameters, and integration strategies in the BCI model including reaching bias in Experiment 1. (**a**) Reaching error according to discrepancy. Solid lines represent the mean behavioral data for each age group, and dotted lines show the corresponding simulation results based on each participant’s best-fitting strategy. The positive and negative values along the vertical axis respectively indicate rightward and leftward reaching errors from the target position; the positive and negative values along the horizontal axis respectively indicate that the visual hand was presented to the right or left of the participant’s proprioceptive hand position. The shaded areas show 95% bootstrap confidence intervals for the behavioral data, and the error bars denote standard errors of the mean. For visibility, data points for the two age groups are slightly offset in the lateral direction. (**b**) Estimated inverse values of visual and proprioceptive sensory reliabilities ($\:{\sigma\:}_{v}$ and $\:{\sigma\:}_{p}$, respectively), and (**c**) estimated prior probability of integrating visual and proprioceptive signals ($\:{p}_{common}$). Boxplots display the median (horizontal line), upper and lower quartiles (box edges), 1.5 × interquartile range (whiskers), and individual participant values (dots). (**d**) Model frequency for the best-fitting strategy (model averaging (MA), model selection (MS), or probability matching (PM)) for each age group, with horizontal dotted lines indicating chance-level frequencies. **p <.01, ***p <.001.

A two-way mixed-design aligned rank transformation (ART)-analysis of variance (ANOVA) on the reaching errors (Fig. 2a) showed a significant main effect of discrepancy (*F* (4, 172) = 273.99, *p* <.001, *η*_p_^2^ = 0.86), but no significant main effect of age (*F* (1, 43) = 2.05, *p* =.160, *η*_p_^2^ = 0.05). The age × discrepancy interaction was significant (*F* (4, 172) = 32.79, *p* <.001, *η*_p_^2^ = 0.43). Friedman tests confirmed the significant effect of discrepancy for both age groups (*χ*^2^s(4) > 61.28, *p*s < .001), indicating that absolute reaching error increased with the visual–proprioceptive discrepancy.

In younger adults, post-hoc Wilcoxon signed-rank tests with Holm correction showed decreased reaching errors at larger discrepancies, with all comparisons significant except 0 cm vs. 14 cm and 7 cm vs. 14 cm. In older adults, the error at 14 cm was smaller than those at all other discrepancies; the error at 7 cm was smaller than that at 0, − 7, and − 14 cm; the error at 0 cm was smaller than that at − 7 and − 14 cm; and the error at − 7 cm was smaller than that at − 14 cm. These patterns indicate that vision shifted the perceived hand position, producing reaching errors opposite the direction of visual-hand displacement. Wilcoxon rank-sum tests showed that older adults exhibited larger absolute reaching errors than younger adults at the ± 7 and ± 14 cm discrepancies, indicating a stronger age-related visual bias consistent with prior work^9,10^.

#### Results of BCI modeling

Table 1 presents the model fit indices. Although the reaching bias-free BCI model fit the data reasonably well, adding each participant’s baseline reaching bias to the model improved its explanatory power and produced lower Bayesian Information Criterion (BIC) values across all strategy comparisons. The summed BIC values obtained for younger adults using the MA, MS, and PM strategies were 4581, 4579, and 4577, respectively, in the model without bias and 3796, 3803, and 3803, respectively, in the model with bias; those obtained for older adults using the MA, MS, and PM strategies were 5387, 5400, and 5402, respectively, in the model without bias and 4894, 4910, and 4909, respectively, in the model with bias. These results show that the MA strategy considering reaching bias provided the best fit in both age groups. A chi-square test of independence showed no significant difference between the strategy frequencies for the two age groups (Fig. 2d; *χ*^²^(2) = 0.79, *p* =.464).

Next, we analyzed the estimated visual variance ($\:{\sigma\:}_{v}$), proprioceptive variance ($\:{\sigma\:}_{p}$), and integration bias (or prior belief regarding causality, $\:{p}_{common}$) parameters for each participant from the best-fitting bias-included BCI model. A two-way ART-ANOVA on sensory variance (Fig. 2b) revealed a significant main effect of sensory modality (*F* (1, 43) = 113.61, *p* <.001, *η*_p_² = 0.73) and no significant main effect of age (*F* (1, 43) = 0.49, *p* =.487, *η*_p_² = 0.01). However, the age × sensory modality interaction was significant (*F* (1, 43) = 37.26, *p* <.001, *η*_p_² = 0.46). Wilcoxon signed-rank tests showed that $\:{\sigma\:}_{p}$ was smaller than $\:{\sigma\:}_{v}$ in both age groups (younger: *V* = 210, *p* <.001, Cliff’s *d* = 1.00; older: *V* = 276, *p* =.001, Cliff’s *d* = 0.68), indicating that proprioception was generally more reliable for hand localization in the considered situation. Wilcoxon rank-sum tests further showed that older adults exhibited a smaller $\:{\sigma\:}_{v}$ than younger adults (*W* = 417, *p* <.001, Cliff’s *d* = 0.67), but a larger $\:{\sigma\:}_{p}$ (*W* = 99, *p* <.001, Cliff’s *d* = 0.60). These results suggest that aging is associated with reduced proprioceptive reliability and relatively increased visual reliability. Note that the estimated $\:{\sigma\:}_{p}$ was significantly correlated with independently-measured unisensory proprioceptive variance (see the Supplementary Information for details). Finally, a Wilcoxon rank-sum test showed a significant age-related increase in $\:{p}_{common}$ (Fig. 2c), with older adults more strongly integrating visual and proprioceptive cues (*W* = 162.5, *p* =.002, Cliff’s *d* = 0.35).

**Table 1:** Comparison of models without and with the estimation of reaching bias in Experiment 1.

Age group	Without reaching bias		With reaching bias		Group-wise BIC without–with reaching bias	Log10(BF)
	Nagelkerke’s *R*^2^		Nagelkerke’s *R*^2^			
	Mean ± SE	CI (95%)	Mean ± SE	CI (95%)		
Younger	.934 ± .014	(.901, .956)	.979 ± .002	(.974, .983)	774.9	168
Older	.957 ± .007	(.938, .967)	.978 ± .002	(.974, .980)	492.1	107

Goodness-of-fit for the BCI models was assessed for each participant using their best-fitting strategy (model averaging, model selection, or probability matching) within each BCI model (without or with reaching bias) across age groups. The coefficient of determination (*R*²) was calculated following Nagelkerke^26^. Group-wise BIC was computed by summing the individual BIC values for each participant’s best strategy, then subtracting the total BIC for the model with reaching bias from that for the model without reaching bias; positive values indicate a better fit for the former, whereas negative values favor the latter. Similarly, positive and negative Log10 Bayes factor (BF) values reflect better group-level fits for the models with and without reaching bias, respectively. Finally, SE indicates standard error and CI indicates the confidence interval.

### Experiment 2

#### Behavioral data

**Fig. 3:**
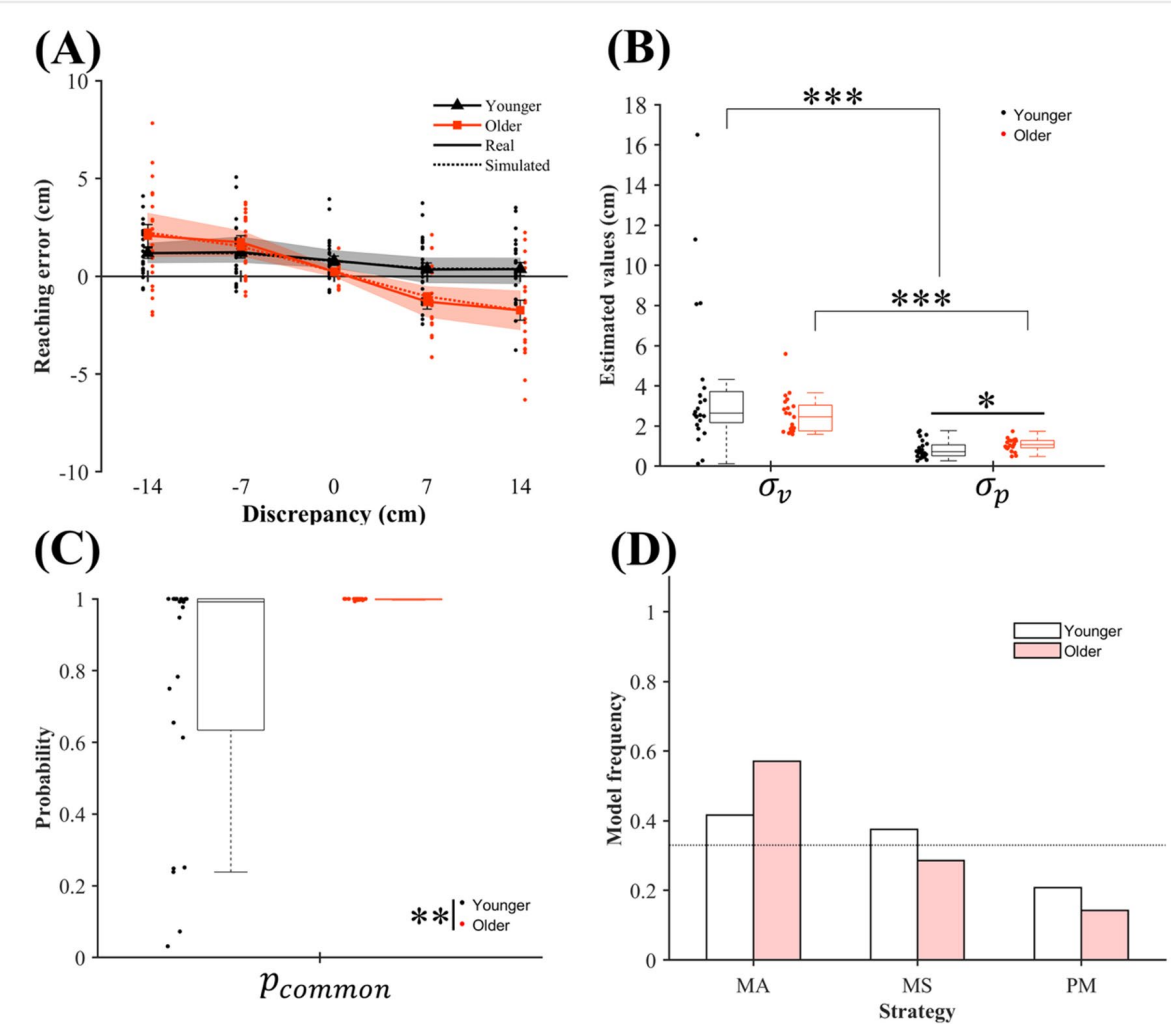
Behavioral results, estimated parameters, and integration strategies in the BCI model including reaching bias in Experiment 2. (**a**) Reaching error according to discrepancy. Solid lines represent the mean behavioral data for each age group, and dotted lines show the corresponding simulation results based on each participant’s best-fitting strategy. The positive and negative values along the vertical axis respectively indicate rightward and leftward reaching errors; the positive and negative values along the horizontal axis respectively indicate that the visual hand was presented to the right or left of the participant’s proprioceptive hand position. The shaded areas show 95% bootstrap confidence intervals for the behavioral data, and the error bars denote standard errors of the mean. For visibility, data points for the two age groups are slightly offset in the lateral direction. (**b**) Estimated inverse values of visual and proprioceptive sensory reliabilities ($\:{\sigma\:}_{v}$ and $\:{\sigma\:}_{p}$, respectively), and (**c**) estimated prior probability of integrating visual and proprioceptive signals ($\:{p}_{common}$). Boxplots display the median (horizontal line), upper and lower quartiles (box edges), 1.5 × interquartile range (whiskers), and individual participant values (dots). (**d**) Model frequency for the best-fitting strategy (model averaging (MA), model selection (MS), or probability matching (PM)) for each age group, with horizontal dotted lines indicating chance-level frequencies. *p <.05, **p <.01, ***p <.001.

A two-way mixed-design ART-ANOVA conducted on the reaching errors (Fig. 3a) showed a significant main effect of discrepancy (*F* (4, 172) = 39.71, *p* <.001, *η*_p_² = 0.48) and no significant main effect of age (*F* (1, 43) = 1.91, *p* =.174, *η*_p_² = 0.04). The age × discrepancy interaction was significant (*F* (4, 172) = 14.56, *p* <.001, *η*_p_² = 0.25). Friedman tests confirmed the significant effect of discrepancy in both age groups (χ²s(4) > 37.32, *p*s < .001), showing that absolute reaching error increased with discrepancy magnitude. In younger adults, post-hoc Wilcoxon signed-rank tests with Holm correction showed smaller errors at 14 cm than at all other discrepancies except 7 cm, at 7 cm than at 0 cm or below, and at 0 cm than at − 7 cm and − 14 cm; the − 14 cm vs. − 7 cm and 7 cm vs. 14 cm contrasts were non-significant. In older adults, errors at 14 cm was smaller than those at all other discrepancies, those at 7 cm was smaller than those at 0 cm or below, and those at 0 cm was smaller than those at − 7 cm and − 14 cm; only the –14 cm vs. –7 cm comparison was non-significant. These post-hoc patterns indicate that vision-biased reaching occurred in both age groups.

Wilcoxon rank-sum tests further showed smaller errors in older than younger adults at 7 and 14 cm, suggesting greater visual weighting in older adults’ hand position estimation. Together with Experiment 1, these results support a stronger age-related visual bias in proprioceptive localization, which is consistent with the results reported in previous studies^9^^,10^.

#### Results of BCI modeling

Table 2 shows that the BCI models with and without reaching bias both adequately explained the individual data. As in Experiment 1, the bias-included model showed better fit than the bias-free model, and the BIC comparisons confirmed this observation. The summed BIC values obtained for younger adults using the MA, MS, and PM strategies were 4947, 4948, and 4950, respectively, in the model without bias and 3981, 3986, and 3987, respectively, in the model with bias; those obtained for older adults using the MA, MS, and PM strategies were 4393, 4395, and 4398, respectively, in the model without bias and 3926, 3935, and 3936, respectively, in the model with bias. These results indicate that the MA strategy with reaching bias best explained the data in both groups, replicating the results of Experiment 1. A chi-square test of independence showed no significant difference between the strategy frequencies for the two age groups (Fig. 3d; *χ*^²^(2) = 0.75, *p* =.748).

Next, we analyzed the estimated parameters ($\:{\sigma\:}_{v}$, $\:{\sigma\:}_{p}$, and $\:{p}_{common}$) from each participant’s best-fitting bias-included BCI model. A two-way ART-ANOVA on sensory variance (Fig. 3b) revealed a significant main effect of sensory modality (*F* (1, 43) = 114.17, *p* <.001, *η*_p_² = 0.73) and no significant main effect of age (*F* (1, 43) = 2.48, *p* =.123, *η*_p_² = 0.05). The significant age × sensory modality interaction (*F* (1, 43) = 8.03, *p* =.007, *η*_p_² = 0.16) prompted post-hoc testing. Wilcoxon signed-rank tests showed that $\:{\sigma\:}_{p}$ was smaller than $\:{\sigma\:}_{v}\:$in both groups (younger: *V* = 202, *p* <.001, Cliff’s *d* = 0.83; older: *V* = 230, *p* <.001, Cliff’s *d* = 0.90), indicating higher proprioceptive reliability. Wilcoxon rank-sum tests showed that younger adults exhibited a smaller $\:{\sigma\:}_{p}\:$than older adults (*W* = 132, *p* =.042, Cliff’s *d* = 0.40), whereas no differences between the $\:{\sigma\:}_{v}\:$of the two age groups were found (*W* = 245, *p* =.372, Cliff’s *d* = 0.20), suggesting that age-related changes in proprioceptive reliability, rather than visual reliability, drive differences in hand localization when visual cues are brief (0.5 s). Finally, $\:{p}_{common}$ showed a significant difference according to age (*W* = 109.5, *p* =.007, Cliff’s *d* = 0.50), with older adults exhibiting a stronger tendency to integrate visual and proprioceptive signals (Fig. 3c). This result aligns with that of Experiment 1 and supports the identified age-related changes in multisensory integration.

**Table 2:** Comparison of models without and with the estimation of reaching bias in Experiment 2.

Age group	Without reaching bias		With reaching bias		Group-wise BIC without–with reaching bias	Log10(BF)
	Nagelkerke’s *R*^2^		Nagelkerke’s *R*^2^			
	Mean ± SE	CI (95%)	Mean ± SE	CI (95%)		
Younger	.949 ± 0.013	(.911, .968)	.984 ± .003	(.976, .989)	963.7	209
Older	.963 ± 0.006	(.950, .972)	.981 ± .002	(.977, .984)	459.2	100

Goodness-of-fit of the BCI models was assessed for each participant using the best-fitting strategy (model averaging, model selection, or probability matching) within each BCI model (without or with reaching bias) across age groups. The coefficient of determination (*R*²) was computed following Nagelkerke^26^. Group-wise BIC was computed by summing the individual BICs for each participant’s best strategy, then subtracting the total BIC for the model with bias from that for the model without bias; positive values indicate a better fit for former, whereas negative values favor the latter. Similarly, positive and negative Log10 Bayes factor (BF) values respectively reflect better group-level fits for the models with and without reaching bias. Finally, SE indicates standard error and CI indicates the confidence interval.

#### Comparisons of estimated parameters between experiments

The comparison analysis was conducted on the dataset limited to participants who completed both experiments and whose BCI model parameter estimates met the inclusion criteria. This resulted in a final sample of 14 younger adults and 18 older adults. The estimated parameters ($\:{\sigma\:}_{v}$, $\:{\sigma\:}_{p}$, and $\:{p}_{common}$) from each participant’s best-fitting bias-included BCI model were statistically compared across experiments. A three-way ART-ANOVA on sensory variance revealed significant main effects of experiment (*F* (1, 90) = 9.05, *p* =.003, *η*_p_² = 0.09) and sensory modality (*F* (1, 90) = 203.44, *p* <.001, *η*_p_² = 0.69). Significant experiment × sensory modality (*F* (1, 90) = 41.11, *p* <.001, *η*_p_² = 0.31) and age × sensory modality (*F* (1, 90) = 28.87, *p* <.001, *η*_p_² = 0.24) interactions were also observed. Other main effects or interactions were non-significant (age group: *F* (1, 30) = 0.59, *p* =.449, *η*_p_² = 0.02; experiment × age: *F* (1, 90) = 1.01, *p* =.319, *η*_p_² = 0.01; experiment × age × modality: *F* (1, 90) = 0.81, *p* =.370, *η*_p_² = 0.01). For the experiment × sensory modality interaction, Wilcoxon signed-rank tests showed that $\:{\sigma\:}_{v}$ was smaller in Experiment 1 than in Experiment 2 (*V* = 50, *p* <.001, Cliff’s *d* = 0.69), whereas $\:{\sigma\:}_{p}$ was larger in Experiment 1 than in Experiment 2 (*V* = 482, *p* <.001, Cliff’s *d* = 0.69). These results suggest reduced reliance on vision in Experiment 2. Other simple effects matched the individual findings of each experiment and are omitted here.

For the age × sensory modality interaction, Wilcoxon rank-sum tests showed that younger adults tended to exhibit a larger $\:{\sigma\:}_{v}$ than older adults (*W* = 171, *p* =.091, Cliff’s *d* = 0.36), indicating a trend toward weaker visual reliance. Younger adults also exhibited a smaller $\:{\sigma\:}_{p}$ than older adults (*W* = 35, *p* <.001, Cliff’s *d* = 0.63), indicating stronger proprioceptive reliance.

When evaluating $\:{p}_{common}$, a two-way ART-ANOVA revealed significant main effects of experiment (*F* (1, 30) = 17.00, *p* <.001, *η*_p_² = 0.36), age (*F* (1, 30) = 8.77, *p* =.006, *η*_p_² = 0.23), as well as a significant experiment × age interaction (*F* (1, 30) = 15.97, *p* <.001, *η*_p_² = 0.35). Wilcoxon signed-rank tests of this interaction showed that both younger and older adults exhibited a weaker $\:{p}_{common}\:$in Experiment 2 than in Experiment 1 (younger: *V* = 59.5, *p* =.018, Cliff’s *d* = 0.50; older: *V* = 55, *p* =.005, Cliff’s *d* = 0.56), suggesting that the manipulation reduced the causal prior for integrating visual and proprioceptive signals.

## Discussion

In this study, both younger and older adults showed systematic reaching errors that increased with spatial discrepancy. Older adults displayed a stronger visual bias than younger adults, which is consistent with previous findings^7–9,^^10^. The BCI model captured these patterns in both groups, showing that older adults’ hand position perception involved reduced proprioceptive reliability and a higher prior for a common source, with no age-related differences in decision strategy. The difference between prior belief according to age group was especially apparent in Experiment 2, when removing the embodiment phase reduced younger adults’ priors but left older adults’ higher priors unchanged. These findings suggest intrinsic age-related differences in causal priors for VP integration that are not driven by repeated exposure to displaced visual feedback.

Various RHI-related studies—including applications of the rubber hand, mirror hand, and projected hand illusions—have consistently shown that perceived hand position shifts toward the visually defined location when visual and proprioceptive inputs conflict; this phenomenon is known as proprioceptive drift^1^. These illusions are typically induced through prolonged synchronous stimulation of real and artificial hands, and perceived hand position is measured by pointing with the unaffected hand or indicating the index finger’s location on a ruler. When participants actively induce an illusion through tapping, reaching, or pointing with the affected hand, perceived position also shifts toward the visual hand^3^. Consistent with this observation, the participants in our study exhibited reaching errors opposite the visual hand across age groups. However, the results of prior studies conducted with older adults (> 65 years) often indicated no differences in drift according to age^4,5,^^6^, whereas our results did. This discrepancy may reflect methodological differences, particularly related to how the illusion was induced and measured. Prior work predominantly used passive induction, whereas we used active induction (Experiment 1) and active measurement (Experiments 1 and 2), which engage different neural processes and can produce different outcomes^3^. Notably, two other studies using active paradigms also found age-related differences^7^^,8^. Overall, these findings point to an age-related increase in visual reliance for body-position perception.

Recent BCI studies have also reported age-related differences in VP integration for hand position perception but attributed older adults’ stronger visual dominance solely to reduced proprioceptive precision^7^^,8^. In contrast, our results indicate that both reduced proprioceptive reliability and an increased $\:{p}_{common}$ contribute to the stronger visual bias observed in older adults, suggesting that age-related multisensory changes involve both sensory reliability and causal inference mechanisms. To our knowledge, this is the first evidence that aging is associated not only with changes in sensory reliability but also with changes in causal priors for VP hand position perception. One methodological difference is that we compared three decision strategies—MA, MS, and PM—and selected each participant’s best-fitting strategy when estimating $\:{p}_{common}$, whereas prior studies relied only on MA. However, in our data, most participants could be described using MA or MS, and parameter estimates from MS were nearly identical to those from MA, indicating that strategy differences are unlikely to account for the discrepancies across studies. A second difference involves the employed hand images. Prior studies used a virtual hand (avatar), whereas we presented a prerecorded image of each participant’s own hand. Critically, RHI research has shown that higher visual realism strengthens embodiment, and object realism can modulate the common-source prior^16^. Thus, a more realistic hand could be expected to increase $\:{p}_{common}$. However, our data do not support this conclusion: Martinelli et al.^7^ reported $\:{p}_{common}$ values above 0.85 for most participants, but approximately half of the younger adults in our study exhibited $\:{p}_{common}\:$values below 0.80, even with a more realistic image.

A more plausible explanation involves the visual-hand exposure duration. Prior studies displayed the virtual hand for 1.5 s, whereas our Experiment 2 only showed the visual hand for 0.5 s, following Fang et al.^16^. Mirror hand illusion research has shown that reaching errors increase with longer exposure^27^, implying that prolonged viewing elevates the probability of assuming a common cause even when sensory precision is unchanged. Consistent with this implication, both Experiment 1 (with longer exposure and active movement) and our preliminary study showed larger $\:{p}_{common}\:$values with longer presentations. These findings suggest that exposure duration may explain the higher causal priors in younger adults reported by Martinelli et al.^7^ and Risso et al.^8^, reducing the differences between younger and older adult groups.

Although many studies have reported enhanced multisensory integration with aging^19^^,20^, debate remains regarding whether this results solely from reduced unisensory reliability—consistent with inverse effectiveness^28^—or from age-related changes in the integration process itself^29^. Aging increases neural noise, lowering sensory representation reliability across modalities, including proprioception. However, enhanced integration has been observed even when stimuli are well above threshold^20^, suggesting additional mechanisms. Our BCI results support this view, indicating that both unisensory and multisensory inference processes change with age, especially in body-related perception. The observed increase in the common-source prior also raises new questions. Does it serve as a compensatory mechanism for degraded unisensory inputs, or does it reflect reduced attentional control, i.e., a diminished capacity to selectively attend to one modality while suppressing another^19^? Future studies should disentangle these possibilities to better understand how aging reshapes causal inference in multisensory perception.

It should be noted that the estimated proprioceptive precision was lower than visual precision in both age groups, which appears inconsistent with some previous behavioral findings. However, this pattern is consistent with previous BCI modeling studies^7^^,8^, in which relative precision estimates reflect task-dependent weighting of sensory information rather than baseline sensory acuity. We speculate that this discrepancy is largely attributable to the experimental procedure. In our task, the visual hand disappeared before the reaching response, and in Experiment 2, its presentation duration was limited to 0.5 s. These factors likely reduced the effective reliability of visual information at the time of action, thereby altering the relative precision estimates derived from the model. Relatedly, one might question why the estimated visual precision was higher in older adults than in younger adults, particularly given the well-established decline in visual acuity with aging. We propose that this finding reflects age-related differences in the flexibility of sensory weighting. Previous studies have shown that older adults tend to continue relying on visual information even after it has disappeared^9^^,30^ or become clearly unreliable^31^, indicating reduced adaptability in reweighting sensory cues. In line with this interpretation, the present results suggest that the internal representation of the visual hand persisted longer in older adults, whereas younger adults may have downweighted or discarded the visual signal once it was no longer available. Consequently, the BCI model may attribute relatively higher visual precision to older adults, capturing differences in sensory persistence or weighting dynamics rather than differences in visual acuity per se.

Previous work has shown that the BCI model explains audiovisual spatial integration in both younger and older adults, demonstrated that age-related declines in auditory reliability—not changes in common-source priors—account for older adults’ performance, and identified age-related differences in causal-inference strategies, with younger adults tending to use PM and older adults relying more on MS^22^. By contrast, our study found that MA was the dominant strategy in both age groups. From a computational perspective, MA is optimal for minimizing the expected squared error. This discrepancy raises a critical question: what explains the difference between causal inference strategies for audiovisual and VP integrations? One possibility is the nature of each sensory source: audiovisual integration typically involves dynamic external events, whereas VP integration involves more stable internal bodily signals. Such differences in environmental variability may shape the adopted strategies. Future work should examine how the statistical properties of sensory contexts influence causal inferences across domains accordingly.

Regarding the neural mechanisms of body perception, RHI studies have implicated the posterior parietal cortex—including the supramarginal gyrus—as well as the temporoparietal junction in hand position perception^32^^,33^. In nonhuman primates, electrophysiological work has shown that premotor neurons encode a common-source probability during hand positioning tasks^16^, and this activity decreases when a wooden arm replaces the projected arm, reinforcing its role in common-source inference. Neuroimaging studies using the BCI model have also shown that posterior parietal activity correlates with model-predicted body-ownership strength^15^. Together, these findings suggest that posterior parietal and premotor cortices support VP integration and likely contribute similarly to the body perceptions of older adults.

This study was subject to several limitations. First, we indirectly assessed the age-related influences of visual information on perceived hand position through reaching errors. Such effects may not appear in perceptual reports, which are more commonly used in aging research based on the RHI (e.g., Campos et al.^4^). Although Holmes et al.^11^ showed similar visual effects on action and perception in mirror-hand contexts, whether this similarly holds in our setup is unclear. Therefore, future work should directly compare action-based and perceptual measures to evaluate the generalizability of these findings. Second, because the participants kept their eyes open during the reaching task, visual reference cues (e.g., display frames) may have influenced localization performance and may have affected the BCI modeling to some extent. This issue is difficult to eliminate in tasks requiring participants to reach toward a visual target, because some visual reference is inherently present whenever a visual stimulus is displayed. However, this potential influence is not specific to our paradigm and is likely present in previous experimental paradigms, including prior BCI studies using reach-to-visual-target tasks^7^^,8^^,16^. Having participants perform the task with eyes closed would eliminate visual reference cues but would introduce an additional confound, as participants would need to rely on spatial memory of the target location. This is particularly problematic in aging studies, given well-documented age-related declines in memory function, and future studies should address this issue. Third, although our modeling approach identified the computational mechanisms underlying stronger visual influence in older adults, several participants were excluded as outliers owing to extreme parameter estimates, suggesting that the BCI model may not fully characterize all individuals. The types of outliers differed rather between age groups. Among older adults, the exclusions likely reflect the distributional properties of the parameter estimates and the applied statistical threshold rather than qualitatively different integration behavior. Among younger adults, in contrast, excluded participants tended to show relatively weak or minimal visual-hand effects, which resulted in unstable variance estimates and distorted model parameter estimation. Overall, these patterns suggest that the excluded participants may reflect individual differences in multisensory integration rather than primarily reflecting data quality issues. Future studies should consider refined models or procedures to accommodate such individual differences and capture the full range of integration behavior.

In conclusion, older adults rely more on visual cues than younger adults when integrating visual and proprioceptive signals for hand position, at least in action-based tasks. The BCI modeling conducted in this study suggests that this shift reflects both reduced proprioceptive reliability and a stronger prior for a common source.

### Methods

#### Participants

Experiment 1 comprised 28 younger adults (8 men and 20 women; mean age ± standard deviation (SD): 21.2 ± 0.94 years) and 28 healthy older adults (12 men and 16 women; mean age ± SD: 76.8 ± 3.64 years). Experiment 2 comprised 28 younger adults (7 men and 21 women; mean age ± SD: 20.9 ± 1.07 years) and 28 healthy older adults (13 men and 15 women; mean age ± SD: 77.3 ± 3.67 years). Note that 20 younger and 25 older adults who participated in Experiment 1 also participated in Experiment 2. The sample size was based on a previous study^22^ that examined age-related differences in BCI model parameters for audiovisual integration in auditory localization. Although that study examined a different sensory domain, it was a valuable reference as the only prior work to investigate age-related differences in sensory reliability, causal prior, and integration strategy using BCI modeling. Potential outliers were compensated for by recruiting additional participants, increasing the sample size to 28 per group compared to 24 in the referenced study. The younger adults were undergraduate and graduate students at Kumamoto University. The older adults were community-dwelling individuals recruited via the Kumamoto City Senior Citizens’ Employment Guidance Center. All participants received monetary compensation. All participants were unaware of the experiment purpose.

Visual acuity was assessed binocularly at distances of 0.4 m (near) and 3.0 m (far) using Landolt C charts, with appropriate vision correction applied. In Experiment 1, older adults had a mean (± SD) visual acuity of 0.60 (± 0.20) for near and 0.71 (± 0.27) for far distances; in Experiment 2, their visual acuity was 0.60 (± 0.20) for near and 0.73 (± 0.28) for far distances. Younger adults had a far-distance visual acuity of 1.22 (± 0.35) in Experiment 1 and 1.25 (± 0.27) in Experiment 2; near-distance vision was not assessed. Mild visual impairment (e.g., macular degeneration, cataracts, or glaucoma) was self-reported by six (Experiment 1) and seven (Experiment 2) older adults. All participants reported intact tactile sensation and no vestibular dysfunction. Older adults reported no neurological, psychiatric, or orthopedic conditions and were not undergoing neuroleptic treatment. Their Mini-Mental State Examination scores exceeded 24 (mean ± SD: 29.0 ± 1.4 in Experiment 1; 29.0 ± 1.5 in Experiment 2). A handedness questionnaire^34^ revealed that 23 and 26 younger adults in Experiments 1 and 2, respectively, and all older adults were right-handed.

This study was approved by the Ethics Committee of the Faculty of Humanities and Social Sciences, Kumamoto University and conducted in accordance with the Declaration of Helsinki (1964). Written informed consent was obtained from all participants prior to participation.

### Apparatus and stimuli

Figure 1a shows the setup and procedures used in Experiments 1 and 2. Visual stimuli were presented on a large, upward-facing display (approximately 70 cm wide × 40 cm high; resolution: 1920 × 1080 pixels; sampling rate: 60 Hz; P3222QE, Dell Inc.) positioned 8 cm above the desk surface using metal brackets. Beneath the display, a sliding board supported the participant’s left hand, allowing lateral movement along a slide rail. A wooden bar aligned with the participant’s left index finger was attached to the board. The lateral position of the bar was tracked in real time using an infrared touch frame (approximately 74 cm wide × 44 cm high; resolution: 1920 × 1080 pixels; sampling rate: 450 Hz; model: ZC32USB10P; Guangzhou Zhanchu Technology Co., Ltd.) placed vertically beside the display.

Each participant’s left-hand image was captured using a webcam (G980, Logitech) beforehand, and a background-removed image of the hand was displayed at its actual size against a gray background. The hand image on the display shifted laterally in real time, synchronized with the participant’s actual hand movements, which were tracked using the infrared touch-frame sensor that recorded position coordinates. The expected maximum temporal discrepancy between event occurrence and visual display update was approximately 28.9 ms, calculated based on the infrared sensor latency (10 ms), its sampling rate (450 Hz), and the monitor refresh rate (60 Hz). The impression that the participant’s real hand was projected onto the tabletop screen was enhanced by fully occluding their real left hand and arm using black paper, which also concealed the underlying apparatus. All stimulus presentations and data collection were controlled using MATLAB R2022b (MathWorks, Natick, MA; https://www.mathworks.com/) with Psychophysics Toolbox extensions (Psychtoolbox-3.0.19; https://psychtoolbox.org/)^35–37^ on a laptop computer (P5-RT-ADLABW11; MouseComputer Co., Ltd.).

### Procedures

Figure 1b and c show the trial sequences for Experiments 1 and 2, respectively. Experiment 1 was followed by Experiment 2 for all participants. First, an image of each participant’s left hand was captured using a webcam. Participants subsequently sat with their midsagittal plane aligned to the center of the tabletop display, resting their left hand on the board beneath the display while viewing the screen above.

#### Experiment 1

We modified the traditional mirror hand illusion paradigm, which elicits embodiment through the active movement of the participant’s hand^11,27^ to support BCI modeling. At the beginning of each trial, the experimenter placed the participant’s hand such that their index finger aligned with one of three lateral locations: either the midsagittal plane (0 cm) or ± 5 cm therefrom (with negative values indicating leftward shifts from the participant’s perspective). A white fixation cross appeared at the center of the display for 1 s. After it disappeared, a visual hand and a black vertical probe line were presented. The visual hand position was at − 14, −7, 0, 7, or 14 cm relative to the actual hand position, and the black line was located 5 cm further to the left of the visual hand position. The participant was instructed to move their left hand laterally until the index finger of their visual hand image was aligned with the black line. Once aligned, the black line shifted sequentially by + 10, −10, and + 10 cm relative to its current position. The participant continued to move their hand to match each new location while maintaining a natural and consistent speed. Following this sequence, the black line shifted again by − 5 cm, prompting the participant to return their hand to the original starting position. The sequence was identical across all starting positions. Once the final alignment was achieved, both the black line and visual hand disappeared, and a red vertical line was presented in the midsagittal plane. Although the final real hand position was not explicitly recorded as an outcome variable, the task was programmed such that the target phase did not begin unless the participant’s real hand position was aligned with the midsagittal plane.

Each participant was instructed to ignore the position of their visual hand after it disappeared and rely on the proprioceptive sense of their left hand to align the center of their index finger with the red line as quickly and accurately as possible. If, when the red line appeared, they believed that the tip of their index finger was already directly beneath the line without moving their left hand, they were instructed not to move it. After completing the reaching movement, the participant pressed a button as quickly as possible with their right hand to record their reaction time (RT), and the final position of their left index finger was captured using the infrared system. If the visual hand had no effect on the perceived hand position, the reaching error would be close to zero; if it influenced the perception, an error in the direction opposite to the visual displacement would be observed.

Participants completed a total of 62 trials. The primary conditions evaluated comprised five visual hand displacements (− 14, − 7, 0, 7, and 14 cm), with the actual hand always starting from the 0 cm position; each condition was repeated ten times. Catch trials with the visual hand at − 7, 0, or 7 cm and the actual hand starting at ± 5 cm positions were included to prevent participants from noticing that there were actually no misalignments between the target line and the actual hand during the main trials. Each catch trial was repeated twice. All trials were conducted in a randomized order. Experiment 1 lasted approximately 20 min per participant.

#### Experiment 2

We adopted a paradigm inspired by previous BCI-modeling studies (e.g., Fang et al.^16^) to clarify intrinsic age-related differences in causal priors. The embodiment phase used in Experiment 1 was omitted as it could otherwise elevate those priors and induce adaptation to the displaced visual feedback. The procedure was identical to that used in Experiment 1 except for this omission: the visual hand appeared statically for 0.5 s before being replaced by the target red line.

### Statistical analyses

The MATLAB R2020b software (MathWorks, Natick, MA; https://www.mathworks.com/) was used for behavioral data processing and plotting. The BCI model parameters were fit to the experimental data using custom Python programs (Python 3.11.9) incorporating PyBADS 1.0.5^38^, a Python implementation of the Bayesian Adaptive Direct Search algorithm^39^. The Python environment was managed using the Anaconda 2024.3-0.3 distribution. Statistical analyses were conducted using JASP 0.17.3 (JASP Team, 2024) and R version 4.2.0 (R Core Team, 2022).

### Behavioral data

Coordinate data were converted to centimeters; the midsagittal plane was set at 0 cm, with leftward positions defined as negative values. The reaching error in each trial was calculated as the horizontal distance between the reach endpoint and target line, with positive and negative reaching errors respectively indicating leftward and rightward shifts in perceived hand position.

Participants for whom the parameters estimated by the best-fitting BCI model (see below) exceeded three times the interquartile range (based on boxplot criteria) were excluded as outliers. This criterion was applied separately to each estimated parameter, and participants were excluded if they met this criterion for any parameter. Based on these criteria, eight younger and three older adults in Experiment 1 and four younger and six older adults in Experiment 2 were excluded (see the Supplementary Information for analyses including the outliers). Thus, 20 younger adults (2 men and 18 women; mean age ± SD: 21.2 ± 1.0 years) and 25 older adults (9 men and 16 women; mean age ± SD: 76.7 ± 3.6 years) were included in the final analysis of Experiment 1. Of these participants, 18 younger adults and all older adults were right-handed. In Experiment 2, one older adult who was unable to complete the task as well as the 10 outliers were excluded. Thus, 24 younger adults (6 men and 18 women; mean age ± SD: 20.8 ± 1.0 years; 14 participants overlapped with Experiment 1) and 21 older adults (8 men and 13 women; mean age ± SD: 77.3 ± 3.7 years; 16 participants overlapped with Experiment 1) were included in the final analysis. Of these participants, 22 younger adults and all older adults were right-handed. In both experiments, participants who were not right-handed were included in the analyses, as their data were consistent with those of right-handed participants.

Shapiro–Wilk tests conducted on reaching errors revealed normality violations in several conditions (younger adults in both experiments, older adults in Experiment 1). Therefore, a two-way ANOVA was performed on the ART data (factors: age × discrepancy). If the age × discrepancy interaction was significant, post-hoc Friedman tests were followed by Wilcoxon signed-rank or rank-sum tests with Holm correction, as appropriate.

Previous research has shown that the influence of vision on the mirror hand illusion diminishes over time once the visual hand representation is removed^9^^,10^^,40^ likely because perceived hand position is recalibrated through afferent proprioceptive signals. This effect was examined by calculating the mean RT corresponding to each discrepancy for each participant. Shapiro–Wilk tests indicated violations of normality in several conditions for younger adults in both experiments. Thus, RT data were analyzed using ART followed by a two-way ANOVA (age × discrepancy). When the interactions were significant, post-hoc analyses were conducted using Friedman tests with Wilcoxon signed-rank or rank-sum tests (Holm correction). The RT data are analyzed in the Supplementary Information.

### BCI model fitting and parameter data analyses

In the BCI model, multisensory signals (e.g., vision and proprioception) are assumed to originate from either the same source ($\:C=1$) or different sources ($\:C=2$). This model uses prior beliefs about causal structure to determine whether the input signals are integrated or processed separately in the human brain. True signal locations (*S*) are unobservable; only noisy measurements (*X*) are available, from which the model infers estimated locations (*Ŝ*). The model infers the causal structure *C* using the following formulation^12^^,13^:

$$\:P\left(C|{X}_{v},\:{X}_{p}\right)=\frac{P\left({X}_{v},\:{X}_{p}|C\right)P\left(C\right)}{P\left({X}_{v},\:{X}_{p}\right)}$$

where $\:{X}_{v}\:$and $\:{X}_{p}$ reflect the internal noisy representations of the visual and proprioceptive hand stimuli, respectively.

In the same-source case $\:P(C=1)$, the true location $\:S$ of the visual and proprioceptive stimuli is assumed to be the same and drawn from a uniform distribution. In the different-sources case $\:P(C=2)$, the true visual and proprioceptive stimuli locations (denoted as $\:{S}_{v}$ and $\:{S}_{p}$, respectively) are assumed to be independently drawn from uniform distributions. Visual and proprioceptive sensory noises are modeled as the SDs of independent Gaussian distributions and given by $\:{\sigma\:}_{v}$ and $\:{\sigma\:}_{p}$, respectively. Under $\:P(C=1)$, the inferred true location is given by the reliability-weighted average of $\:{X}_{v}\:$and$\:\:{X}_{p}$^18^ as follows:

$$\:{\widehat{S}}_{p,\:C=1}=\:{\widehat{S}}_{v,\:C=1}=\frac{\frac{{X}_{p}}{{\sigma\:}_{p}^{2}}+\:\frac{{X}_{v}}{{\sigma\:}_{v}^{2}}}{\frac{1}{{\sigma\:}_{p}^{2}}+\:\frac{1}{{\sigma\:}_{v}^{2}}}$$

For the different sources case $\:P(C=2)$, the optimal estimates of the true locations are given by

$$\hat{S}_{{p,~C = 2}} = X_{p} ,~~\hat{S}_{{v,~C = 2}} = X_{v}$$

A decision-making strategy must be subsequently specified to estimate the final proprioceptive location. Previous studies have proposed three such strategies: model averaging (MA), model selection (MS), and probability matching (PM)^12^^,13^^,21^.

In the MA strategy, the final estimate minimizes the expected mean squared error and represents the average of the two causality estimates ($\:C=1$ and $\:C=2$) weighted by their posterior probabilities as follows:

$$\:{\widehat{S}}_{p}=\:P\left(C=1|{X}_{v},\:{X}_{p}\right){\widehat{S}}_{p,\:C=1}+(1-P\left(C=1|{X}_{v},\:{X}_{p}\right)){\widehat{S}}_{p,\:C=2}$$

In the MS strategy, the final estimate is the one corresponding to the more probable causal structure as follows:

$$\:{\widehat{S}}_{p}=\left\{\begin{array}{c}{\widehat{S}}_{p,\:C=1}\:\:if\:P\left(C=1|{X}_{v},\:{X}_{p}\right)>\:0.5\\\:{\widehat{S}}_{p,\:C=2}\:\:if\:P\left(C=1|{X}_{v},\:{X}_{p}\right)\le\:0.5\end{array}\right.$$

Finally, in the PM strategy, the final estimate is chosen stochastically by sampling a random value ($\:\alpha\:$) from a uniform [0, 1] distribution on each trial as follows:

$$\:{\widehat{S}}_{p}=\left\{\begin{array}{c}{\widehat{S}}_{p,\:C=1}\:\:if\:P\left(C=1|{X}_{v},\:{X}_{p}\right)>\:\alpha\:\\\:{\widehat{S}}_{p,\:C=2}\:\:if\:P\left(C=1|{X}_{v},\:{X}_{p}\right)\le\:\:\alpha\:\end{array}\right.$$

The BCI model described above consisted of three free parameters: $\:{\sigma\:}_{v}$, $\:{\sigma\:}_{p}$, and the prior belief regarding causality ($\:{p}_{common}\:\equiv\:\:P(C=1)$). Notably, we did not include a spatial prior (as used in other studies^12^^,22^) because previous research on hand position perception has not reported a centralization bias, i.e., a systematic tendency to shift responses toward the center of the presented spatial range.

Previous research has shown that the proprioceptively perceived hand position can shift laterally leftward for the left hand and rightward for the right hand, resulting in a rightward reaching bias when the left hand is used to reach a target^41^. We accounted for this shift by incorporating the reaching bias ($\:rb$) as an additional free parameter in a modified version of the BCI model, adding it to the final proprioceptive estimate $\:{\widehat{S}}_{p}$. Thus, the extended, bias-included model considered four free parameters: $\:{\sigma\:}_{v}$, $\:{\sigma\:}_{p}$, $\:{p}_{common}$, and $\:rb$. We estimated these parameters for each participant in both BCI model versions (with and without bias).

The reaching errors were converted to the estimated perceived finger (or hand) positions by reversing the number signs to compare model responses and behavioral data. The reaching plane in front of the participant was divided into 41, 1 cm long bins in the lateral direction for computation purposes such that the observed error was rounded to one of 41 perceived positions instead of an infinite number of possible positions. The model responses were binned in the same manner. The expected model response for each stimulus input $\:P\left({\widehat{S}}_{p}\right|{S}_{v},\:{S}_{p})$ was approximated by repeatedly sampling $\:{X}_{v}\:$and $\:{X}_{p}$ from Gaussian distributions $\:N({S}_{v},\:{\sigma\:}_{v})$ and $\:N({S}_{p},\:{\sigma\:}_{p})$, respectively, then obtaining the model response from $\:{X}_{v}\:$and $\:{X}_{p}$. This procedure was repeated 10,000 times. Subsequently, we set the minimum value of 0.01 for the model response probabilities, when the probability was below that value, and renormalized the distribution. We defined the likelihoods of the 41 bins of behavioral data using a multinomial distribution with the assumed true probabilities as the simulated model responses $\:P\left({\widehat{S}}_{p}\right|{S}_{v},\:{S}_{p})$. The fitted model parameters were obtained for each participant, decision-making strategy, and BCI model by maximizing the likelihood of the behavioral data given the model parameters. The optimization was repeated 100 times with random initial values to find the best-fit parameters. The lower and upper bound settings for PyBADS were $\:\{{\sigma\:}_{v},\:{\sigma\:}_{p},\:{p}_{common}\}=\{0,\:0,\:0\}$ and $\:\{100,\:100,\:1\}$, respectively, for the BCI model without reaching bias, and $\:\{{\sigma\:}_{v},\:{\sigma\:}_{p},\:{p}_{common},\:rb\}=\{0,\:0,\:0,\:-35\}$ and $\:\{100,\:100,\:1,\:35\}$, respectively, for the BCI model with reaching bias. The plausible lower and upper bound settings were $\:\{{\sigma\:}_{v},\:{\sigma\:}_{p},\:{p}_{common}\}=\{0.01,\:1,\:0.01\}$ and $\:\{10,\:30,\:0.99\}$, respectively, for the BCI model without reaching bias, and $\:\{{\sigma\:}_{v},\:{\sigma\:}_{p},\:{p}_{common},\:rb\}=\{0.01,\:1,\:0.01,\:-35\}$ and $\:\{10,\:30,\:0.99,\:35\}$, respectively, for the BCI model with reaching bias. Initial values were sampled uniformly within these plausible ranges.

Models were compared using $\:BIC=klogN+2NLL\left(\widehat{\theta\:}\right)$, where *k* is the number of parameters, *N* is the number of trials, and *NLL* is the negative log-likelihood at the best-fit parameter $\:\widehat{\theta\:}$. The group-level BIC was obtained by summing the individual BICs within each age group. The models with and without reaching bias were also compared using the approximated Bayes factors from BIC differences as $\:B{F}_{01}=exp(\varDelta\:BI{C}_{10}/2)$, where $\:B{F}_{01}$ is the Bayesian factor for hypothesis (model) $\:{H}_{0}$ relative to $\:{H}_{1}$, and $\:\varDelta\:BI{C}_{10}=BIC\left({H}_{1}\right)-BIC\left({H}_{0}\right)$^42^. The goodness-of-fit of each model was assessed using Nagelkerke’s$\:\:R^{2} = \left( {1 - \exp \left( { - \frac{2}{n}\left( {NLL_{0} - NLL_{M} } \right)} \right)} \right)/\left( {1 - \exp \left( { - \frac{2}{n}NLL_{0} } \right)} \right)$, where $\:{NLL}_{M}$ is the negative log-likelihood of the model and $\:{NLL}_{0}$ is the negative log-likelihood of the null model^26^. The null model was defined as having a flat response distribution. Consequently, the model with reaching bias explained the behavioral data better than the model without it (see the Results section).

Age-related differences between strategies (MA, MS, and PM) were investigated by calculating the relative summed BIC values in the reaching bias-included models for each age group and statistically analyzing them using a chi-square test of independence (strategy × age group). The BCI model has a nonlinear probabilistic structure and includes parameters with bounded ranges (e.g., $\:{p}_{common}$ ∈ [0, 1]; σ ≥ 0), which can produce non-normal parameter distributions. Therefore, we adopted non-parametric statistical tests for the estimated parameters. Specifically, all estimated parameters were analyzed using the ART method. Sensory reliability was examined using a two-way ART-ANOVA (factors: age × modality). When a significant age × modality interaction was found, post-hoc comparisons were conducted using Wilcoxon signed-rank or rank-sum tests, as appropriate. Wilcoxon rank-sum tests were conducted for $\:{p}_{common}$ in both experiments.

Finally, the estimated parameters were compared across experiments. Sensory reliability and integration bias were examined via a three-way ART-ANOVA (factors: experiment × age × modality) and two-way ART-ANOVA (factors: experiment × age), respectively. Significant interactions were followed by post-hoc Wilcoxon signed-rank or rank-sum tests, as appropriate.

## Supplementary Information

Below is the link to the electronic supplementary material.

Supplementary Material 1

## Data Availability

Raw (anonymized) data supporting the results of the present study are available in the Open Science Framework at https://osf.io/84fzq/. The analysis codes are available from the corresponding author on reasonable request.
