# Supplementary material for: Bayesian causal inference reveals declined proprioception, increased integration bias underlie older adults’ stronger visual bias in hand position perception
Source: Sci Rep. 2026 Apr 12;16:17048. doi: 10.1038/s41598-026-45797-3 (PMC13230816; doi:10.1038/s41598-026-45797-3)
Supplement: Supplementary file 1 — Supplementary Material 1 [file 41598_2026_45797_MOESM1_ESM.pdf]

Supporting Information for:

Bayesian Causal Inference Reveals Declined Proprioception, Increased Integration Bias Underlie Older Adults' Stronger Visual Bias in Hand Position Perception

Naoki Kuroda[1,2,3,*], Yoshiyuki Sato[4], Shinya Harada[3], Ryo Teraoka[5], and Wataru Teramoto[3,*]

[1] Research Organization of Open Innovation and Collaboration, Ritsumeikan University, 2-150 Iwakura-cho, Ibaraki, Osaka 567-8570, Japan

[2] Japan Society for the Promotion of Science, Kojimachi Business Center Building, 5-3-1 Kojimachi, Chiyoda-ku, Tokyo 102-0083, Japan

[3] Faculty of Humanities and Social Sciences, Kumamoto University, 2-40-1 Kurokami, Kumamoto, 860-8555 Japan

[4] Faculty of Human Informatics Department of Human Informatics, Aichi Shukutoku University, 2-9, Katahira, Nagakute-city, Aichi, 480-1197, Japan

[5] Graduate School of Engineering, Muroran Institute of Technology, 27-1 Mizumoto-cho, Muroran, Hokkaido, 050-8585, Japan

*Corresponding authors

Email: n.kuroda25@gmail.com (Naoki Kuroda)

Email: teramoto@kumamoto-u.ac.jp (Wataru Teramoto)

Introduction

This supplementary information presents the results of an additional experiment investigating whether the estimated proprioceptive variance obtained through the Bayesian causal inference (BCI) model matched the actual proprioceptive variance in the proprioceptive-pointing task without vision, the reaction time (RT) data analyses for Experiments 1 and 2, and the results of additional analyses including the outlier participant data, which were excluded from the analyses presented in the main text, using the same analysis methods.

Validity of estimated proprioceptive variance in the BCI model

We confirmed model reliability by conducting an additional session in Experiment 1 after all other sessions were completed. In this session, a red target line appeared at the midsagittal plane after fixation disappeared. Participants were asked to align their real index finger with the target as quickly and accurately as possible. They were also instructed to identify their actual left-hand position without moving their hand if they already perceived their real index finger to be at target position when the target was presented. The session comprised 22 trials: 10 main trials with the actual hand starting at the 0 cm position and 12 catch trials with the actual hand starting at ±5 cm positions

(6 trials started at –5 cm and 6 at 5 cm). Catch trials were included to prevent participants from understanding that the correct answer was not to move the hand in the main trials. The standard deviation of the actual left hand positions in the main trials for each participant, except for outliers, was compared with the estimated proprioceptive variance (σ_p) in the reaching bias-included BCI model using Pearson's correlation analysis for each age group because the Shapiro–Wilk tests revealed no violations of the normality assumption. If the reaching bias-included BCI model is able to estimate the correct proprioceptive variance, a positive correlation should be observed between the standard deviation of proprioceptive position in the additional session and the proprioceptive variance estimated by the reaching bias-included BCI model in each age group.

The correlation analysis revealed a significant positive correlation in each age group (younger: $r = .65$, $p = .002$, 95% bootstrap confidence interval (CI) [.34, .87]; older: $r = .62$, $p = .001$, 95% bootstrap CI [.29, .81]) (**Figure S1**). These results suggest that parameter estimation in the reaching bias-included BCI model was successful.

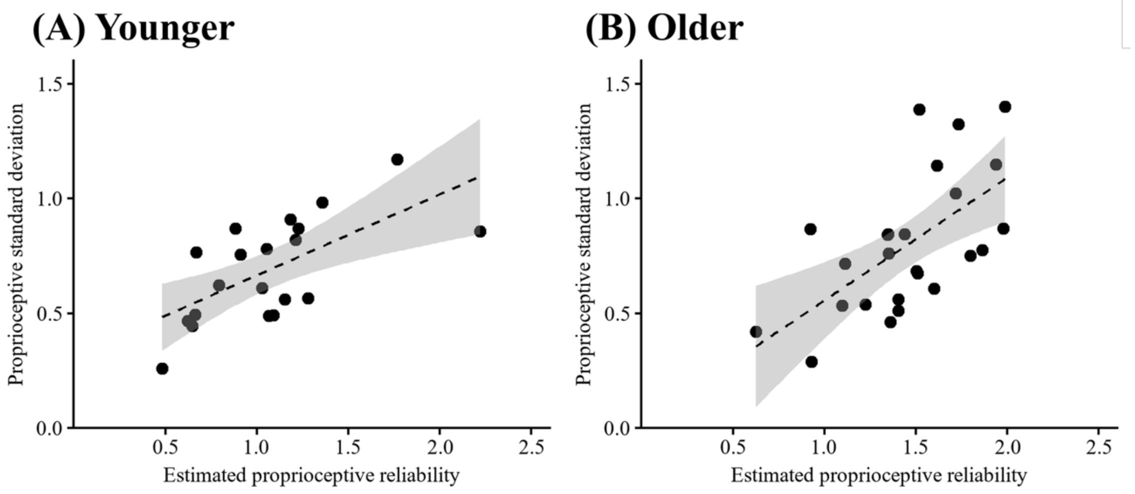

Figure S1. Results of Pearson's correlation analyses for (a) younger and (b) older groups in Experiment 1. The dots denote individual data points. Larger positive values along the horizontal and vertical axes indicate lower proprioceptive reliability. The gray area denotes a 95% confidence interval.

RT data analyses in each experiment

Figure S2 shows the RT data from each experiment. In Experiment 1, a two-way ART-ANOVA showed significant main effects of age (F (1, 43) = 10.52, p = .002, η_p^2 = 0.20) and discrepancy (F (4, 172) = 29.09, p < .001, η_p^2 = 0.40). Older adults exhibited slower RTs than younger adults. In terms of discrepancy, Holm-corrected comparisons showed slower RTs at larger discrepancies, with no differences between –14 and 14 cm or –7 and

7 cm. The age × discrepancy interaction was not significant (F (4, 172) = 1.39, p = .240, η_p^2 = 0.03).

In Experiment 2, a two-way ART-ANOVA showed significant main effects of age (F (1, 43) = 8.65, p = .005, η_p^2 = 0.17) and discrepancy (F (4, 172) = 10.55, p < .001, η_p^2 = 0.20), as well as a significant interaction between the two (F (4, 172) = 7.68, p < .001, η_p^2 = 0.15). Wilcoxon rank-sum tests indicated that older adults exhibited slower RTs than younger adults at −14, −7, and 14 cm, with no differences at 0 or 7 cm. Friedman tests showed discrepancy effects within each age group (χ^2s (4) > 10.76, ps < .030). For older adults, signed-rank tests with Holm correction showed slower RTs at –14, –7, and 14 cm than at 0 cm, and faster RTs at 7 cm than at –14 and 14 cm. No pairwise differences were observed for younger adults. These findings indicate that the older adults' stronger visual bias was not a result of faster responses, as their RTs were slower overall.

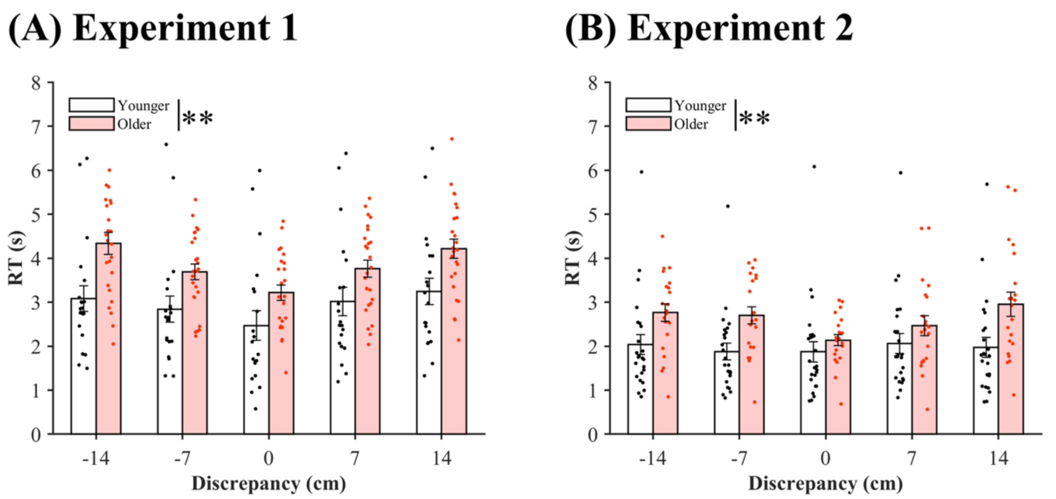

Figure S2. RT results for (a) Experiment 1 and (b) Experiment 2. The bars show the mean RT for each discrepancy condition in each age group, and the dots show individual data points. The error bars denote standard errors of the mean. $**p < .01$.

Data analyses including outliers

Participants

Experiment 1 comprised 28 younger adults (8 men and 20 women; mean age ± standard deviation (SD): 21.2 ± 0.94 years) and 28 community-dwelling older adults (12 men and 16 women; mean age ± SD: 76.8 ± 3.64 years). Of these participants, 23 younger adults and all older adults were right-handed. Experiment 2 comprised 28 younger adults (7 men and 21 women; mean age ± SD: 20.9 ± 1.07 years) and 28 community-

dwelling older adults (13 men and 15 women; mean age ± SD: 77.3 ± 3.67 years); data from 27 older adults (12 men and 15 women; mean age ± SD: 77.2 ± 3.71 years) were included in the analyses due to one participant being unable to complete the task. Of these participants, 26 younger adults and all older adults were right-handed. In both experiments, participants who were not right-handed were included in the analyses, as their data were consistent with those of right-handed participants.

Results

Experiment 1: Behavioral data

A two-way mixed-design ART-ANOVA on the reaching errors (Figure S3a) revealed a significant main effect of discrepancy (F (4, 216) = 222.30, p < .001, η_p^2 = 0.80), but no significant main effect of age (F (1, 54) = 2.99, p = .090, η_p^2 = 0.05). The age × discrepancy interaction was significant (F (4, 216) = 26.18, p < .001, η_p^2 = 0.33). A Friedman test confirmed the significant effect of discrepancy for both age groups (χ^2s (4) > 74.91, ps < .001), indicating that absolute reaching error increased with the absolute value of the visual–proprioceptive discrepancy.

In younger adults, post-hoc Wilcoxon signed-rank tests with Holm correction showed increased reaching errors at larger discrepancies, with all pairwise comparisons significant except 7 cm vs. 14 cm. In older adults, the same tests revealed that all

pairwise comparisons were significant. These comparisons suggest that vision systematically shifted the perceived hand position, producing reaching errors in the direction opposite the visual hand in both age groups. Regarding the age effect, Wilcoxon rank-sum tests showed that older adults exhibited significantly larger absolute reaching errors than younger adults at both ±7 and ±14 cm discrepancies. These results indicate a stronger visual bias in hand position perception with age, which is consistent with previous research[1,2].

Experiment 1: Results of BCI modeling

The relative summed BIC values obtained for younger adults using the model averaging (MA), model selection (MS), and probability matching (PM) strategies were 6554, 6547, and 6547, respectively, in the model without bias and 5548, 5557, and 5558, respectively, in the model with bias; those obtained for older adults using the MA, MS, and PM strategies were 6106, 6115, and 6118, respectively, in the model without bias and 5534, 5553, and 5553, respectively, in the model with bias. These results indicate that using the MA strategy considering reaching bias provided the best fit in both age groups. A chi-square test of independence revealed no significant difference between the strategy frequencies for the two age groups (Figure S3d; χ^2 (2) = 0.75, p = .580).

Next, we analyzed the estimated the estimated visual variance (σ_v),

proprioceptive variance (σ_p), and integration bias (or prior belief regarding causality, p_{common}) for each participant from their best-fitting BCI model including reaching bias. A two-way ART-ANOVA for sensory variance (Figure S3b) revealed significant main effects of age (F (1, 54) = 5.14, p = .027, η_p^2 = 0.09) and modality (F (1, 54) = 63.75, p < .001, η_p^2 = 0.54), as well as a significant age × modality interaction (F (1, 54) = 22.19, p < .001, η_p^2 = 0.29). Simple main effect analyses conducted using Wilcoxon signed-rank tests showed that σ_p was significantly smaller than σ_v in both age groups (younger: V = 380, p < .001, Cliff's d = 0.86; older: V = 344, p = .001, Cliff's d = 0.64), indicating that proprioception was generally more reliable than vision for hand localization. Wilcoxon rank-sum tests further revealed that older adults exhibited a significantly smaller σ_v than younger adults (W = 647, p < .001, Cliff's d = 0.65); by contrast, they exhibited a significantly larger σ_p (W = 216, p = .003, Cliff's d = 0.45). Considering these effect sizes, aging appears to be associated with decreased proprioceptive reliability and relatively increased visual reliability in hand localization. Finally, a Wilcoxon rank-sum test showed a significant age-related increase in p_{common} (Figure S3c), with older adults more strongly integrating visual and proprioceptive cues (W = 223.5, p < .001, Cliff's d = 0.43).

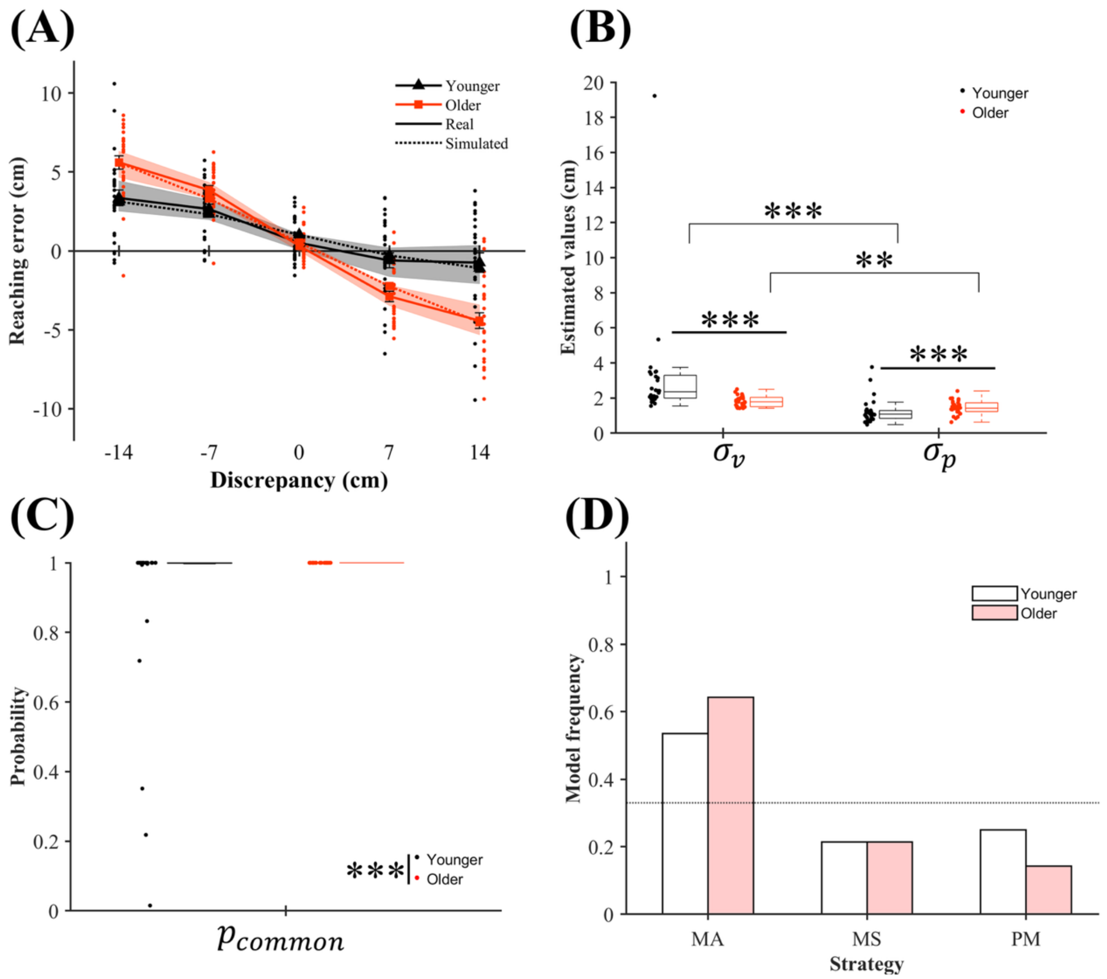

Figure S3. Behavioral results, estimated parameters, and integration strategies in the BCI model including reaching bias in Experiment 1. (a) Reaching error according to discrepancy. Solid lines represent the mean behavioral data across participants for each age group, and dotted lines show the corresponding simulation results based on each participant's best-fitting strategy. The positive and negative values along the vertical axis respectively indicate rightward and leftward reaching errors from the target position; the positive and negative values along the horizontal axis respectively indicate

that the visual hand was presented in right-side and left-side positions from the participant's proprioceptive hand position. The shaded areas indicate 95% bootstrap confidence intervals for the behavioral data, and the error bars show the standard errors of the mean. For visibility, data points from the two age groups are slightly offset in the lateral direction. (b) Estimated inverse values of the sensory reliabilities of vision and proprioception (σ_v and σ_p, respectively), and (c) estimated prior probability of integrating the visual and proprioceptive signals (p_{common}). The boxplots display the median (horizontal line), upper and lower quartiles (box edges), 1.5 × interquartile range (whiskers), and individual participant values (dots). (d) Model frequency for the best-fitting strategy (model averaging (MA), model selection (MS), or probability matching (PM)) for each age group, with the horizontal dotted lines indicating chance-level frequencies. $^{**}p < .01$, $^{***}p < .001$.

Experiment 2: Behavioral data

A two-way mixed-design ART-ANOVA on the reaching errors (Figure S4a) revealed a significant main effect of discrepancy (F (4, 212) = 32.34, $p < .001$, $\eta_p^2 = 0.38$) and no significant main effect of age (F (1, 53) = 1.03, $p = .316$, $\eta_p^2 = 0.02$), but the age × discrepancy interaction was significant (F (4, 212) = 11.45, $p < .001$, $\eta_p^2 = 0.18$).

Friedman tests confirmed the significant effect of discrepancy in both age groups (χ^2s (4) > 25.1, *p*s < .001), indicating that the absolute reaching error generally increased with the magnitude of discrepancy. In younger adults, post-hoc Wilcoxon signed-rank tests with Holm correction showed that reaching errors were significantly smaller at 0, 7, and 14 cm than at −14 and −7 cm; the other pairs were not significant. In older adults, reaching errors were significantly smaller at 14 cm than at all other discrepancies except for 7 cm, at 7 cm than at 0 cm or below, and at 0 cm than at −7 or −14 cm. These post-hoc results confirm that visual cues systematically biased reaching in both age groups. Wilcoxon rank-sum tests further showed that the absolute reaching errors at 7 and 14 cm were significantly larger in older than younger adults, suggesting that older adults rely more on visual information for hand position estimation. When considered together with the results of Experiment 1, these results reinforce the conclusion that older adults exhibit a stronger visual bias in proprioceptive hand localization, which is consistent with the conclusions of previous research[1,2].

Experiment 2: Results of BCI modeling

Consistent with the results of Experiment 1, the reaching bias-included model provided a better fit than the bias-free model, a conclusion supported by the BIC comparisons.

The relative summed BIC values obtained for younger adults using the MA, MS, and PM strategies were 5781, 8781, and 5783, respectively, in the model without bias and 4600, 4605, and 4608, respectively, in the model with bias; those obtained for older adults using the MA, MS, and PM strategies were 5772, 5772, and 5774, respectively, in the model without bias and 5043, 5053, and 5053, respectively, in the model with bias. These results indicate that the MA strategy considering reaching bias best explained the data for both age groups, confirming the findings from Experiment 1. A chi-square test of independence revealed no significant difference between strategy frequencies for the two age groups (Figure S4d; χ^2 (2) = 0.76, p = .548).

Next, we analyzed the estimated parameters (σ_v, σ_p, and p_{common}) for each participant using their best-fitting BCI model including reaching bias. A two-way ART-ANOVA conducted on sensory variance (Figure S4b) revealed significant main effects of age (F (1, 53) = 9.78, p = .003, η_p^2 = 0.16) and modality (F (1, 53) = 10.27, p = .002, η_p^2 = 0.16). A significant age × modality interaction (F (1, 53) = 9.40, p = .003, η_p^2 = 0.15) prompted post-hoc tests. Wilcoxon signed-rank tests showed that σ_p was significantly smaller than σ_v for both age groups (younger: V = 397, p < .001, Cliff's d = 0.86; older: V = 372, p < .001, Cliff's d = 0.78), indicating higher proprioceptive reliability regardless of age. Wilcoxon rank-sum tests revealed that younger adults

exhibited a significantly smaller σ_p than older adults (W = 230, p = .012, Cliff's d = 0.39), suggesting greater proprioceptive reliability in the former. No significant differences between the σ_v for the two age groups were found (W = 491, p = .057, Cliff's d = 0.30), suggesting that age-related changes in proprioceptive reliability, rather than visual reliability, underlie the observed effects on hand localization when visual cues are brief (0.5 s). Finally, p_{common} showed a significant difference according to age group (W = 228.5, p = .011, Cliff's d = 0.40), with older adults exhibiting a stronger bias toward integrating visual and proprioceptive signals (Figure S4c). This finding is consistent with the results of Experiment 1 and further supports the identified age-related changes in multisensory integration.

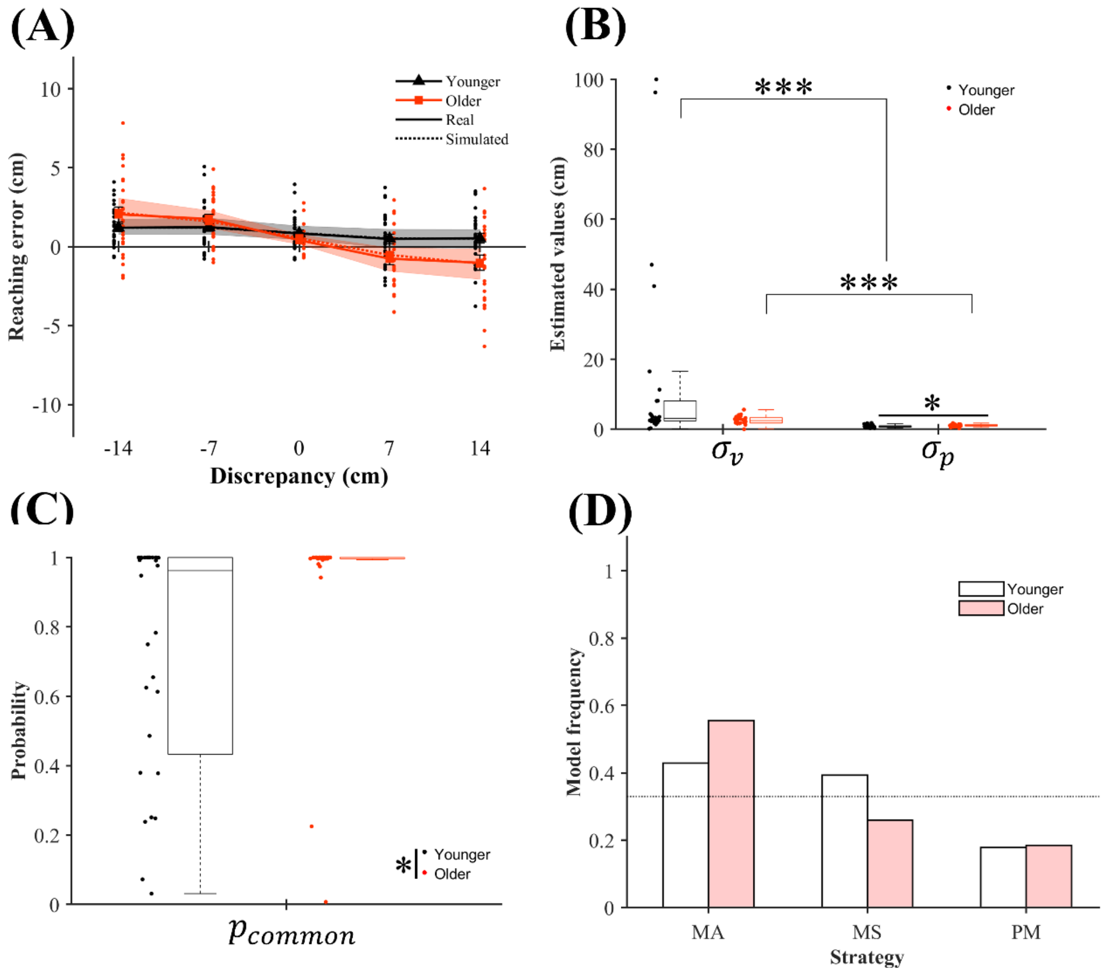

Figure S4. Behavioral results, estimated parameters, and integration strategies in the BCI model including reaching bias in Experiment 2. (a) Reaching error according to discrepancy. Solid lines represent the mean behavioral data across participants for each age group, and dotted lines show the corresponding simulation results based on each participant's best-fitting strategy. The positive and negative values along the vertical axis respectively indicate rightward and leftward reaching errors from the target position; the positive and negative values along the horizontal axis respectively indicate that the visual hand was presented in the right-side and left-side positions from the

participant's proprioceptive hand position. The shaded areas indicate 95% bootstrap confidence intervals for the behavioral data, and the error bars denote the standard errors of the mean. For visibility, data points from the two age groups are slightly offset in the lateral direction. (b) Estimated inverse values of the sensory reliabilities for vision and proprioception (σ_v and σ_p, respectively), and (c) estimated prior probability of integrating the visual and proprioceptive signals (p_{common}). The boxplots display the median (horizontal line), upper and lower quartiles (box edges), 1.5 × interquartile range (whiskers), and individual participant values (dots). (d) Model frequency for the best-fitting strategy (model averaging (MA), model selection (MS), or probability matching (PM)) for each age group, with the horizontal dotted lines indicating chance-level frequencies. *$p < .05$, ***$p < .001$.

Analyses of RT during reaching in each experiment with outliers

Figure S5 shows the RTs obtained during reaching. A two-way ART-ANOVA conducted on the Experiment 1 RTs revealed significant main effects of age (F (1, 54) = 14.41, $p < .001$, $\eta_p^2 = 0.21$) and discrepancy (F (4, 216) = 36.98, $p < .001$, $\eta_p^2 = 0.41$), with older adults exhibiting slower RTs than younger adults. Analyzing the main effect of discrepancy, multiple comparisons with Holm correction showed significantly slower RTs

at larger discrepancies; however, no significant differences were observed between the −14 and 14 cm or −7 and 7 cm conditions. The age × discrepancy interaction was not significant (F (4, 216) = 1.48, p = .209, η_p^2 = 0.03).

A two-way ART-ANOVA on the Experiment 2 RTs revealed significant main effects of age (F (1, 53) = 6.96, p = .011, η_p^2 = 0.12) and discrepancy (F (4, 212) = 13.14, p < .001, η_p^2 = 0.20), as well as a significant age × discrepancy interaction (F (4, 212) = 5.89, p < .001, η_p^2 = 0.10). Analyzing the simple main effect of age, Wilcoxon rank-sum tests showed that older adults exhibited significantly slower RTs than younger adults at −14, −7, and 14 cm, but there were no significant differences at 0 cm and 7 cm. Analyzing the simple main effect of discrepancy, Friedman tests revealed significant differences across discrepancy conditions within each age group (χ^2s (4) > 15.54, ps < .004). For older adults, pairwise comparisons conducted using the Wilcoxon signed-rank test with Holm correction revealed that RTs were significantly slower at −14, −7, and 14 cm than at 0 cm; whereas those at 7 cm were faster than those at −14 and 14 cm (all Holm-corrected). For younger adults, RTs were significantly slower at −14 cm than at −7 and 0 cm; the other pairs were not significant.

These findings indicate that the stronger visual bias exhibited by older adults did not stem from faster responses, as their RTs were generally slower than those of younger

adults.

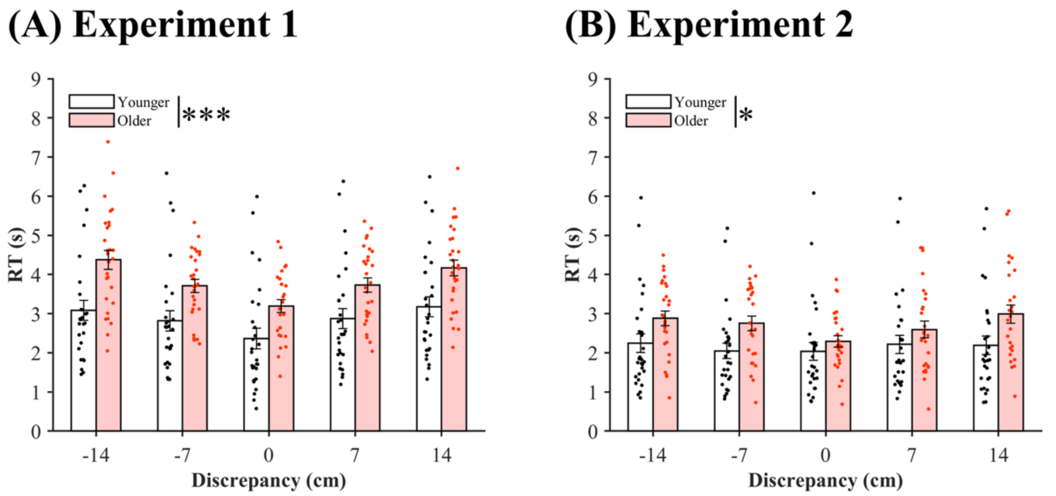

Figure S5. RTs obtained by (a) Experiment 1 and (b) Experiment 2. The bars indicate the mean RT values across all participants in each age group for each discrepancy condition, the dots indicate individual data points, and the error bars denote the standard errors of the mean. *p < .05, ***p < .001.

References

1. Teramoto, W. Age-related changes in visuo-proprioceptive processing in perceived body position. *Sci. Rep.* **12**, 8330 (2022).
2. Teraoka, R., Kuroda, N. & Teramoto, W. Interoceptive sensibility is associated with the temporal update of body position perception. *Psychologia* **65**, 4–16 (2023).
